# Comment on Yoon et al. Heterogeneous Colorectal Cancer Risk in Women with Metabolic Dysfunction-Associated Steatotic Liver Disease by Age, Lipid, and Waist-Circumference: A Nationwide Cohort Study. *Cancers* 2026, *18*, 125

**DOI:** 10.3390/cancers18152446

**Published:** 2026-07-30

**Authors:** Haonan Jin

**Affiliations:** Department of Pharmacy, Shaoxing Central Hospital, Shaoxing University, Shaoxing 312000, China; 531330992@qq.com

The study by Yoon et al. [1] leverages a nationwide prospective cohort of 483,401 Korean women to systematically evaluate the association between metabolic dysfunction-associated steatotic liver disease (MASLD) and colorectal cancer (CRC) risk. Its large scale and robust design provide valuable population-based evidence on the oncological consequences of MASLD. The authors further report that the MASLD-related CRC risk appears particularly elevated among women aged 40–49 years, those without dyslipidemia, and those with a waist circumference < 85 cm. If validated, this finding could offer novel avenues for early identification and risk-stratified management of high-risk subgroups. We highly commend the authors for their significant contribution to the emerging field at the intersection of metabolic liver disease and cancer epidemiology.

That said, after thorough review of the manuscript, we believe several methodological considerations warrant discussion—particularly regarding potential bias in exposure definition and unmeasured confounding. Addressing these points may facilitate more robust interpretation of the current findings and inform methodological refinements in future research.

First, the reliance on the Hepatic Steatosis Index (HSI) to define MASLD may introduce BMI-dependent selection bias. As described, HSI is calculated as: 8 × (ALT/AST) + BMI + 2 (female) + 2 (if diabetic). A threshold of >36 is used to indicate hepatic steatosis. While HSI offers operational convenience in large-scale epidemiological studies, its linear incorporation of BMI may lead to differential misclassification across body size strata. For example, a woman with a BMI of 30 could be classified as having MASLD even with a low ALT/AST ratio (<0.7), whereas a lean woman (e.g., BMI = 22) would require an ALT/AST ratio exceeding 1.5 to surpass the same threshold. Importantly, an elevated ALT/AST ratio typically reflects active hepatocellular injury or inflammation rather than isolated steatosis. Consequently, the “lean MASLD” subgroup may be inadvertently enriched with individuals harboring underlying inflammatory liver conditions—a known driver of carcinogenesis. Thus, the heightened CRC risk observed in the waist circumference < 85 cm subgroup may reflect the pro-oncogenic effects of chronic liver inflammation rather than an intrinsic risk specific to the non-obese phenotype. This apparent heterogeneity could partly stem from a classification artifact inherent to the mathematical structure of HSI, rather than true biological variation.

Second, the null association between MASLD and CRC risk among women with dyslipidemia (HR = 0.99) compared to the elevated risk in those without dyslipidemia (HR = 1.15) suggests potential indication bias. In the Korean National Health Insurance Service (NHIS) system, a diagnosis of dyslipidemia is strongly associated with statin prescription. Accumulating evidence—including multiple meta-analyses—indicates that statins confer a 10–20% reduction in CRC risk through chemopreventive mechanisms [2]. However, the present analysis adjusts for dyslipidemia only as a binary covariate (or incorporates it into the MASLD definition) without directly accounting for statin exposure (e.g., duration, dosage, or adherence). It is therefore plausible that the protective effect of statins has attenuated or masked the underlying MASLD-associated CRC risk in the dyslipidemia group, while the elevated risk observed in the non-dyslipidemia group more closely approximates the natural history of MASLD in the absence of pharmacological intervention. Integrating prescription records to adjust for actual statin use would help disentangle the independent carcinogenic effect of MASLD.

Third, the analysis may not have fully adjusted for several key confounders, notably family history of CRC and use of aspirin or nonsteroidal anti-inflammatory drugs (NSAIDs)—both well-established modulators of CRC risk [3,4]. Although the NHIS database captures prescription medications comprehensively, over-the-counter aspirin use and detailed family history are likely underascertained or incompletely recorded. If the distribution of these factors differs systematically across MASLD subgroups (e.g., by obesity status or dyslipidemia), residual confounding could bias effect estimates and compromise internal validity. Future studies might mitigate this limitation by linking to supplementary data sources (e.g., national health screening questionnaires or cancer registries) or conducting sensitivity analyses under plausible assumptions about unmeasured confounding.

In light of these considerations, we respectfully suggest that the authors consider sensitivity analyses in future work—for instance, by adjusting for statin use duration and intensity, and by redefining MASLD using alternative fatty liver indices less dependent on BMI (e.g., the Fatty Liver Index [FLI] with rigorous stratification). Such approaches would help determine whether the association between “lean” MASLD and elevated CRC risk remains robust after addressing these methodological concerns. Until then, clinicians should interpret the reported risk heterogeneity in non-obese subgroups with appropriate caution.

## Data Availability

No new data were created or analyzed in this study. Data sharing is not applicable to this article.
